# Comparative Cost Evaluation of Managed Entry Agreement Techniques Using Real-World Data from High-Cost Anticancer Drugs in Thailand

**DOI:** 10.3390/jmahp14010017

**Published:** 2026-03-20

**Authors:** Piyapat Owat, Chaoncin Sooksriwong, Hataiwan Ratanabunjerdkul, Tuangrat Phodha

**Affiliations:** 1 Drug Information and Consumer Protection Center, Center of Excellence in Pharmacy Practice and Management Research, Faculty of Pharmacy, Thammasat University, Khlong Luang 12120, Pathum Thani, Thailand; piyapat.owat@dome.tu.ac.th (P.O.); tuangrat25@tu.ac.th (T.P.); 2 Medical Oncology Unit, Department of Internal Medicine, Faculty of Medicine, Thammasat University, Khlong Luang 12120, Pathum Thani, Thailand; rhataiwa@tu.ac.th

**Keywords:** access, budget, cost saving, expenditure, high-cost drug, innovative drug, managed entry agreement, pharmaceutical policy, procurement

## Abstract

High-cost innovative anticancer drugs pose challenges for health systems in balancing timely patient access with long-term financial sustainability. In Thailand, reliance on Health Technology Assessment for reimbursement decisions may delay access, highlighting the potential role of Managed Entry Agreements (MEAs) as complementary policy instruments to manage uncertainty related to price, effectiveness, and use; however, MEA application remains limited and lacks an analytical framework for technique selection. This study used real-world data from Thammasat University Hospital to examine and compare the cost-saving performance of five MEA techniques—discount, free initiation treatment, utilization cap, conditional treatment continuation, and pay-by-result—across six high-cost anticancer drugs representing dominant uncertainty characteristics. Drug procurement costs were modeled over a 24-month horizon from the payer’s perspective, and one-way sensitivity analyses were conducted using ±10% variation in median progression-free survival. Free initiation treatment generated the highest cost savings across uncertainty types, followed by conditional treatment continuation, while utilization cap and discount produced more moderate savings. Pay-by-result demonstrated the lowest cost-saving potential. Sensitivity analyses confirmed the robustness of comparative rankings. Overall, the findings indicate that MEA performance varies according to dominant sources of drug-related uncertainty and support a more structured, context-appropriate approach to MEA selection to strengthen market access and value-based pricing in Thailand.

## 1. Introduction

Thailand’s pharmaceutical expenditure has risen steadily over the past two decades [1], driven largely by the increasing availability of high-cost drugs, particularly targeted anticancer therapies [2]. These trends pose significant challenges for Thailand’s Universal Health Coverage (UHC) system, which aims to ensure equitable access to essential drugs and healthcare services while operating under constrained public budgets [3]. In this context, prioritization is critical for optimizing healthcare resource allocation and safeguarding long-term system sustainability. Consequently, the inclusion of high-cost drugs in the National List of Essential Medicines (NLEM) is guided by rigorous evaluations of clinical effectiveness, safety, cost-effectiveness, and overall value to the health system [4].

Health Technology Assessment (HTA) is a widely recognized policy instrument for priority setting in Thailand, particularly for decisions regarding inclusion in the NLEM and UHC benefit packages [5,6]. Full HTA provides a comprehensive assessment of the clinical, economic, and social implications of health technologies. However, in practice, HTA implementation in Thailand faces structural and procedural challenges that may delay reimbursement decisions. The pathway involves sequential evidence submission, methodological and economic review, multi-stakeholder appraisal, and price negotiation prior to NLEM inclusion. These steps often require repeated data clarification, especially when long-term survival or real-world effectiveness data remain immature at launch. Coordination across regulatory agencies, academic HTA bodies, and payer institutions can further extend review timelines, particularly for high-cost anticancer drugs requiring detailed budget impact assessments. Although these processes ensure methodological rigor and fiscal accountability, they may inadvertently prolong the interval between regulatory approval and reimbursement under UHC [5,6].

As a consequence, prolonged timelines in the HTA process may have important clinical and economic implications. Evidence from Thailand demonstrates that targeted anticancer therapies, such as osimertinib for EGFR-mutation-positive non-small-cell lung cancer (NSCLC), provide meaningful survival benefits [7]. In contexts where effective therapies are available, extended intervals between regulatory approval and reimbursement may therefore limit timely patient access to treatments with established clinical value. Beyond clinical considerations, delayed access can increase downstream healthcare expenditure, out-of-pocket burdens, and inequities across public benefit schemes, while also imposing opportunity costs on the health system [8].

Given these challenges, Managed Entry Agreements (MEAs) have emerged as alternative or complementary instruments to full HTA, particularly for high-cost drugs with uncertainty in price, effectiveness, or use [9]. MEAs are contractual agreements between payers and pharmaceutical companies that specify conditions for reimbursement or coverage to enable earlier patient access while managing financial and clinical risk [10,11]. These agreements are commonly categorized as financial-based agreements, which aim to control prices and budget impact, and performance-based agreements, which address uncertainty related to real-world effectiveness [12]. By improving budget predictability and generating real-world evidence during routine use, MEAs can complement HTA and support subsequent reassessment [8]. Consequently, MEAs have been adopted in several countries as policy tools to facilitate access to high-cost innovative drugs while containing pharmaceutical expenditure [13,14].

In Thailand, the implementation of MEAs remains limited. To date, MEAs have primarily been applied as financial-based agreements delivered through Patient Access Programs (PAPs) for selected high-cost anticancer drugs, typically negotiated on a case-by-case basis with a focus on short-term budget management. Broader adoption—particularly of performance-based agreements—has been constrained by operational and institutional challenges, including the absence of standardized national outcome monitoring systems, limited capacity for data integration across care settings, and administrative difficulties in tracking patient-level outcomes [15]. Consequently, there is increasing recognition of the need for an analytical framework to align specific MEA techniques with dominant sources of drug-related uncertainty—such as price, effectiveness, and use—to support more effective payer–pharmaceutical company negotiations and policy outcomes.

To address this gap, this study aimed to examine and compare the performance of alternative MEA techniques for high-cost anticancer drugs in Thailand under distinct uncertainty conditions. Six high-cost anticancer drugs—pertuzumab, osimertinib, afatinib, ceritinib, palbociclib, and ribociclib—were purposively selected to represent dominant uncertainty, including price, effectiveness, and use. Five MEA techniques—discounts, free initiation treatment, utilization caps, conditional treatment continuation, and pay-by-result—were assessed using real-world drug utilization data to estimate potential drug procurement cost savings from the payer’s perspective [16]. The findings provide empirical evidence to support a more structured and context-appropriate selection of MEA techniques aligned with dominant sources of drug-related uncertainty.

## 2. Materials and Methods

### 2.1. Study Design and Scope

This study employed a retrospective, longitudinal, cost-based economic evaluation using patient-level real-world drug utilization data to assess the cost-saving potential of alternative MEA techniques for high-cost anticancer drugs in Thailand. The analysis was designed to support reimbursement decision-making by comparing procurement cost outcomes under different MEA techniques, rather than to estimate clinical effectiveness or comparative health outcomes.

Patients receiving each study drug were followed over a defined observation period to capture treatment duration, utilization patterns, and associated drug procurement costs under alternative reimbursement scenarios. The longitudinal structure was used to reflect real-world treatment trajectories relevant to payer liability and budget impact, rather than to infer causal clinical relationships.

The analysis consisted of two sequential components. First, drug procurement costs were estimated under five MEA techniques—discount, free initiation treatment, utilization cap, conditional treatment continuation, and pay-by-result—for each study drug, reflecting different sources of reimbursement-related uncertainty, including price, effectiveness, and use. Second, cost outcomes across MEA techniques were compared within each drug to identify which MEA techniques demonstrated greater cost-saving potential under each dominant uncertainty condition.

All analyses were conducted from the payer’s perspective. Patients were followed over a 24-month time horizon, selected to reflect typical treatment durations and reimbursement monitoring periods for high-cost anticancer drugs in the Thai public healthcare system, while ensuring sufficient completeness and consistency of real-world utilization data across study drugs. This time horizon also aligns with practical procurement planning and MEA negotiation cycles within public health benefit schemes.

### 2.2. Population and Sample

The unit of analysis comprised two levels: the drug level and the patient level.

#### 2.2.1. Drug-Level Analysis

At the drug level, the study population consisted of high-cost anticancer drugs used at Thammasat University Hospital (TUH), a tertiary-care public hospital in Thailand. A purposive sampling approach was applied to enable comparative evaluation of MEA techniques under distinct uncertainty conditions, rather than to represent the entire national anticancer portfolio.

Drug selection was guided by three policy-relevant criteria:

1. Relatively high procurement costs with potential budget impact for public payers;
2. Routine use within public health benefit schemes, ensuring availability of real-world utilization data;
3. The presence of a predominant reimbursement-related uncertainty influencing procurement and coverage decisions.

To enhance conceptual clarity, drug classification followed an explicit analytical framework grounded in reimbursement risk theory. Three domains of uncertainty were defined a priori according to their primary implications for payer financial exposure [17]:

- Price uncertainty refers to financial risk driven by high unit prices or substantial cumulative treatment costs that may generate significant and immediate budget impact.
- Effectiveness uncertainty reflects uncertainty regarding real-world or long-term clinical outcomes that may affect value-for-money assessments and reimbursement justification.
- Use uncertainty captures unpredictability in treatment duration, patient eligibility, continuation patterns, or adherence behavior that may lead to divergence between projected and realized budget impact.

Each drug was assigned to the domain representing the principal driver of reimbursement-related financial risk at the time of procurement decision-making in the Thai public healthcare context. The classification was informed by the maturity of clinical evidence, observed variability in treatment duration at TUH, and procurement cost structure.

Accordingly, the selected drugs were categorized as follows:

- Price uncertainty: pertuzumab, osimertinib;
- Effectiveness uncertainty: afatinib, ceritinib;
- Use uncertainty: palbociclib, ribociclib.

This categorization reflects the dominant uncertainty influencing reimbursement exposure under routine practice conditions, while recognizing that multiple uncertainty dimensions may coexist. The purpose of this classification is analytical—to enable structured comparison of MEA techniques across different primary uncertainty drivers—rather than to suggest exclusivity of uncertainty types.

#### 2.2.2. Patient-Level Analysis

At the patient level, the study population included all patients who initiated treatment with one of the six selected high-cost anticancer drugs at TUH between 1 January 2010 and 31 March 2025. Patients were followed longitudinally from treatment initiation until treatment discontinuation, switching to another anticancer therapy, death, or the end of the observation period, whichever occurred first.

To ensure sufficient treatment exposure for modeling real-world drug utilization and procurement costs, patients who had completed fewer than a predefined minimum number of treatment cycles were excluded. These minimum exposure thresholds were operationalized using indication-specific median progression-free survival (PFS)–based treatment durations derived from published evidence (Table 1). The median PFS was applied as a pragmatic reference for the typical treatment duration observed in routine clinical practice and to ensure adequate follow-up for retrospective cost modeling, rather than as a measure of individual clinical benefit.

Because median PFS varies across clinical indications, thresholds were derived from the primary indication for which each drug was most commonly used in routine practice at TUH. This approach allowed exclusion criteria to reflect indication-specific treatment patterns while maintaining internal consistency across drugs and supporting comparability of MEA simulations.

Patients who switched to another anticancer therapy or permanently discontinued treatment were censored at the time of switching or discontinuation. Treatment restarts following discontinuation were treated as separate treatment episodes and were not pooled with the initial treatment course to avoid double-counting of utilization and costs.

The final sample size for each study drug after the application of the exclusion criteria is reported in Section 3. This patient selection approach ensured that the included observations provided sufficient longitudinal data to support a robust cost-based economic evaluation of alternative MEA techniques from the payer’s perspective.

### 2.3. Data Source

Two primary data sources were used. Patient-level utilization data were obtained from the TUH electronic medical record and hospital information systems, including treatment initiation and discontinuation dates, number of treatment cycles administered, and dosing patterns required for procurement cost estimation. Information on treatment response assessed according to RECIST (Response Evaluation Criteria in Solid Tumors) criteria [24] and the occurrence of serious adverse events (grade ≥ 3) [25] was extracted to support the application of selected MEA techniques.

Drug price data were obtained from the Drug and Medical Supply Information Center (DMSIC), which provides official median procurement prices used by public healthcare providers in Thailand. These prices were applied uniformly across all modeled scenarios to reflect real-world reimbursement practices from the payer perspective.

### 2.4. Data Collection

A structured data collection form was developed to support retrospective extraction of patient-level and drug-level information required for the longitudinal, cost-based economic evaluation. The form was designed to capture key variables relevant to reimbursement analysis, including treatment initiation and discontinuation dates, number of treatment cycles administered, dosing patterns, and drug procurement costs, in accordance with the study design, population, and payer perspective.

To ensure relevance and internal consistency of collected variables for economic modeling and MEA simulation, the data collection form was reviewed by three independent experts with complementary expertise in health policy and pharmaceutical reimbursement. The panel included a senior health policy researcher from the Health Systems Research Institute and two academic pharmacists with expertise in pharmacoepidemiology, pharmaceutical pricing, health economics, and reimbursement policy. All items demonstrated acceptable content relevance, with item–objective congruence (IOC) scores of ≥0.67.

Based on expert feedback, minor revisions were made to improve the clarity of variable definitions and alignment with real-world clinical and reimbursement practices, particularly with respect to treatment cycles, treatment discontinuation, and cost-related variables. The finalized form was subsequently used for retrospective data extraction.

Data collection was conducted using electronic medical records and hospital information systems at TUH, in accordance with institutional data governance policies and ethical approval requirements.

### 2.5. MEA Technique Simulation and Scenario Design

Five MEA techniques were simulated based on commonly reported global practices and feasibility within the Thai reimbursement context [16,26]. The objective of the simulation was to assess the cost-saving potential of alternative MEA structures under standardized assumptions, rather than to reproduce specific confidential agreements. All scenarios were applied uniformly across study drugs to enhance comparability.

- **Scenario 1: Discount technique**

A fixed percentage reduction was applied to the unit price of each drug across all treatment cycles. The payer was assumed to reimburse all administered cycles at the discounted price, with no restriction on treatment duration.

- **Scenario 2: Free initiation treatment technique**

The pharmaceutical company was assumed to provide a predefined number of initial treatment cycles free of charge. The number of free cycles corresponded to the median PFS-based treatment duration used as a reference threshold for each drug. The payer reimbursed treatment costs only after completion of the free initiation phase. All subsequent cycles were reimbursed at the full unit price, with no upper limit on treatment duration.

- **Scenario 3: Utilization cap technique**

The payer was assumed to reimburse treatment costs for all treated patients, regardless of clinical response, up to a predefined maximum number of cycles corresponding to the median PFS-based treatment duration for each drug. Treatment cycles exceeding this cap were assumed to be supplied free of charge by the pharmaceutical company. Patients who discontinued treatment before reaching the cap were reimbursed for the actual number of cycles received.

- **Scenario 4: Conditional treatment continuation technique**

Payer reimbursement was conditional on early clinical response. Treatment costs were reimbursed only for patients who demonstrated clinical benefit at a predefined assessment point, as determined according to RECIST criteria. Clinical benefit was defined as achieving complete response (CR), partial response (PR), or stable disease (SD). Patients who did not demonstrate clinical benefit (progressive disease or death) were assumed to discontinue treatment without payer reimbursement. For responding patients, the payer reimbursed treatment costs up to a maximum duration corresponding to the median PFS-based treatment duration, with treatment cycles beyond this point supplied free of charge by the pharmaceutical company.

- **Scenario 5: Pay-by-result technique**

Payer reimbursement was directly linked to treatment outcomes. Treatment costs were reimbursed by the payer only for patients who achieved and maintained a clinical response, as defined according to RECIST criteria (CR, PR, or SD). Patients who failed to achieve clinical response or who discontinued treatment before reaching the median PFS-based duration were assumed not to incur any payer reimbursement, with treatment costs for these non-responding or early-discontinuation cases fully borne by the pharmaceutical company through refunds. For responding patients, the payer was assumed to continue reimbursing treatment costs as long as clinical benefit was maintained, even if treatment duration extended beyond the median PFS-based reference period. No predefined upper limit on treatment duration was imposed; payer liability ended at the time of loss of response, disease progression, or death.

Across all scenarios, median PFS-based treatment duration was used as a pragmatic reference for defining payer liability limits where applicable, enabling consistent comparison of cost outcomes across MEA techniques.

### 2.6. Data Analysis

Data analysis was conducted at the patient level and aggregated to the drug level for comparative assessment. For each study drug and MEA technique, total drug procurement costs were calculated by summing the costs of treatment cycles reimbursed by the payer across all eligible patients during the observation period.

Total procurement cost was calculated as

$$Total\;procurement\;cost=\sum_{i=1}^{n}\left({unit\;price\;per\;cycle\times number\;of\;payer-reimbursed\;cycles}_{i}\right)$$

where *i* denotes individual patients, and the number of reimbursed cycles varied according to the specific MEA structure and scenario assumptions described in the Section 2.5 Treatment cycles supplied free of charge by the pharmaceutical company or refunded under performance-based agreements were excluded from payer cost calculations [27].

Patient-level costs were accumulated over a 24-month time horizon or until treatment discontinuation, switching to another anticancer therapy, death, or censoring, whichever occurred first. Treatment restarts following discontinuation were analyzed as separate treatment episodes and were not pooled with the initial treatment course.

All cost calculations were based on median drug procurement prices obtained from the DMSIC. Costs were initially calculated in Thai Baht and converted to United States dollar (USD) using an exchange rate of 1 USD = 33.6165 Thai Baht [28], consistent with the average exchange rate during the study period.

For each drug, procurement costs under each MEA scenario were compared with a reference case in which no MEA was applied. The following outcomes were reported: (1) mean drug procurement cost per patient, (2) total drug procurement cost, and (3) absolute cost savings compared with the reference case.

Finally, MEA techniques were ranked within each dominant uncertainty category (price, effectiveness, and use) based on observed cost-saving performance to identify which MEA structures demonstrated greater economic value under different uncertainty conditions.

### 2.7. Sensitivity Analysis

One-way sensitivity analyses were performed by varying the median PFS-based reference duration by ±10% for each drug to reflect uncertainty in real-world treatment duration. Variations were applied to MEA scenarios with duration-based payer liability, and their impact on procurement costs and cost savings was examined to assess the robustness of comparative rankings across MEA techniques.

## 3. Results

### 3.1. Patient Demographics

A total of 161 patients were included in this study (Table 2): 13 received pertuzumab, 66 received osimertinib, 9 received afatinib, 11 received ceritinib, 23 received palbociclib, and 39 received ribociclib. The mean age ranged from 59.23 years in the pertuzumab cohort to 71.56 years in the afatinib cohort. Patients receiving pertuzumab, palbociclib, and ribociclib were entirely female, reflecting their approved indications for breast cancer.

Across all six cohorts, the Civil Servant Medical Benefit Scheme (CSMBS) was the most common health benefit scheme. All patients were diagnosed with stage IV disease, consistent with the use of these high-cost targeted and biologic therapies in advanced cancer settings. Biomarker testing confirmed full eligibility for targeted treatment in all cohorts, including HER2 positivity for pertuzumab, EGFR mutation positivity for osimertinib and afatinib, ALK rearrangement for ceritinib, and HER2-negative status for palbociclib and ribociclib.

No formal statistical comparisons of demographic characteristics across drug cohorts were performed, as this study did not aim to compare outcomes between drugs. Patient characteristics are presented descriptively to provide contextual information on the study population, while procurement cost comparisons were conducted within each drug under alternative MEA scenarios.

### 3.2. Cost-Saving Outcomes Under Alternative MEA Techniques

This study evaluated the cost-saving performance of five MEA techniques—discount, free initiation treatment, utilization cap, conditional treatment continuation, and pay-by-result—across six high-cost anticancer drugs representing three dominant types of drug-related uncertainty: price, effectiveness, and use. Cost outcomes were assessed from the payer’s perspective over a 24-month time horizon and are summarized in Table 3.

#### 3.2.1. Drug with Price Uncertainty

For drugs primarily characterized by price uncertainty (pertuzumab and osimertinib), the free initiation treatment technique generated the greatest procurement cost savings, reducing total costs by 72.43% for pertuzumab and 62.80% for osimertinib compared with the reference case. These savings were largely driven by the transfer of early treatment-phase costs to pharmaceutical companies, where a substantial proportion of patients incurred costs during the initial treatment period.

The conditional treatment continuation technique ranked second, yielding moderate but substantial cost savings (53.68% for pertuzumab and 52.90% for osimertinib), reflecting the exclusion of non-responding patients from payer reimbursement. Discount and utilization cap techniques produced more modest yet predictable savings, ranging from 27.57% to 50.00%. In contrast, the pay-by-result technique achieved the lowest cost savings (15.70–26.10%), largely due to the high clinical response rates observed for these drugs, which limited the proportion of costs shifted away from the payer.

#### 3.2.2. Drug with Effectiveness Uncertainty

Among drugs associated with effectiveness uncertainty (afatinib and ceritinib), the free initiation treatment technique consistently demonstrated the highest cost-saving potential, achieving savings of 79.75% and 79.29%, respectively. This reflects the high proportion of early treatment discontinuation or non-response in routine clinical practice, which substantially reduced payer liability under this MEA structure.

The conditional treatment continuation technique ranked second, generating cost savings ranging from 42.86% to 58.23%, as reimbursement was restricted to patients demonstrating clinical benefit. Discount and pay-by-result techniques yielded moderate cost savings. In contrast, the utilization cap technique resulted in the lowest savings in this uncertainty category (approximately 20%), reflecting its limited ability to account for variability in treatment effectiveness beyond the predefined reimbursement threshold.

#### 3.2.3. Drug with Use Uncertainty

For drugs primarily characterized by use uncertainty (palbociclib and ribociclib), the free initiation treatment technique again achieved the greatest cost savings, reducing total procurement costs by 59.87% and 61.35%, respectively. These savings reflect uncertainty in treatment duration and patient continuation patterns, which disproportionately affect early treatment phases.

The conditional treatment continuation technique ranked second, offering balanced payer protection with savings ranging from 52.23% to 61.15%. The utilization cap technique generated moderate but predictable savings (38.65–40.13%), supporting expenditure control by limiting payer liability beyond predefined treatment durations. The pay-by-result technique consistently produced the lowest cost savings in this group (12.10–22.49%), reflecting sustained treatment continuation among responding patients.

#### 3.2.4. Summary of Comparative Performance

Across all three drug-related uncertainty types, the ranking of MEA techniques was consistent. The free initiation treatment technique demonstrated the greatest cost-saving potential across all drug profiles, followed by conditional treatment continuation. Discount and utilization cap techniques provided moderate and predictable savings, while the pay-by-result technique yielded the lowest cost savings under the assumptions applied in this analysis. These findings underscore the importance of aligning MEA design with the dominant source of drug-related uncertainty to maximize economic value from the payer’s perspective.

### 3.3. Sensitivity Analysis: Impact of Changes in Median PFS

One-way sensitivity analyses were conducted by varying the median PFS-based reference duration by ±10% to reflect uncertainty in real-world treatment duration (Table 4). Changes in median PFS differentially affected cost-saving outcomes depending on whether payer liability was explicitly linked to treatment duration.

For the free initiation treatment technique, a 10% increase in median PFS resulted in higher cost savings across all study drugs, as a greater proportion of treatment cycles fell within the pharmaceutically subsidized initiation phase. Conversely, a 10% decrease in median PFS reduced cost savings, reflecting fewer free cycles being utilized before payer reimbursement commenced.

In contrast, the conditional treatment continuation and utilization cap techniques exhibited an inverse pattern. Cost savings declined when median PFS increased, due to longer payer-funded treatment durations before reaching the reimbursement cap or continuation threshold. When median PFS decreased by 10%, cost savings improved as payer liability was limited to shorter treatment courses.

As expected, the discount technique was insensitive to changes in median PFS, as cost savings were applied uniformly on a per-cycle basis and were independent of treatment duration.

Despite variation in absolute cost savings, the ranking of MEA techniques remained unchanged across all sensitivity scenarios. The free initiation treatment technique consistently demonstrated the highest cost-saving potential, followed by conditional treatment continuation. These findings indicate that while duration-based MEA outcomes are sensitive to uncertainty in real-world treatment duration, the comparative performance and policy implications of MEA selection remain robust.

## 4. Discussion

This study provides empirical evidence that the economic performance of MEA techniques varies systematically according to the dominant source of drug-related uncertainty. Using real-world utilization data for six high-cost anticancer drugs in Thailand, the analysis demonstrates that no single MEA technique is universally optimal; rather, cost-saving potential depends on how effectively the MEA structure addresses uncertainty related to price, effectiveness, or use. Across all uncertainty categories, however, the free initiation treatment technique consistently achieved the highest cost savings, followed by conditional treatment continuation.

### 4.1. Mechanisms Underlying Cost Savings Across MEA Techniques

The superior performance of the *free initiation treatment* technique reflects its capacity to shift financial risk away from the payer during the early phase of treatment, when uncertainty regarding continuation and clinical benefit is typically greatest. While the risk-shifting mechanism of such front-loaded agreements is conceptually intuitive, our findings provide empirical quantification of its magnitude under real-world utilization conditions. In this study, the technique generated substantial cost savings for drugs characterized by effectiveness uncertainty (approximately 79%), followed by those with price uncertainty (63–72%), demonstrating that the extent of cost redistribution varies across different uncertainty profiles. These results therefore extend beyond theoretical expectation by illustrating how real-world treatment duration, response patterns, and discontinuation behavior influence the economic performance of this MEA structure. However, despite its economic attractiveness, practical implementation may be constrained, as pharmaceutical companies often limit the number of free cycles offered in real-world negotiations [9,11,16].

The *conditional treatment continuation* technique ranked second in terms of cost savings and performed particularly well for drugs associated with use uncertainty and effectiveness uncertainty. By linking reimbursement to demonstrated clinical benefit, this technique reduces payer expenditure on non-responding patients while maintaining access for those who benefit from treatment. This mechanism mirrors outcome-based reimbursement models implemented in countries such as Italy, where performance-based agreements are supported by national monitoring registries. However, its feasibility depends on the availability of reliable outcome data and administrative capacity to assess treatment response in routine practice [9,14,16].

The *utilization cap* technique produced moderate but predictable savings, particularly for drugs characterized by use uncertainty. This finding is consistent with the widespread use of utilization caps in Thailand’s existing PAPs, where limiting payer liability beyond a predefined threshold helps contain budget impact. While administratively simpler than outcome-based agreements, utilization caps still require accurate tracking of treatment cycles and may impose additional reporting burdens on providers [9,15].

In contrast, the *pay-by-result* technique yielded the lowest cost savings across most drug-related uncertainty categories. High response rates among the studied drugs limited the scope for reimbursement, reducing the financial benefit of this technique. Similar observations have been reported in European oncology settings, where outcome-based agreements generated limited savings when most patients achieved favorable clinical outcomes. These findings highlight that pay-by-result techniques may be less effective when uncertainty is driven more by price or utilization than by effectiveness [9,16].

Finally, the *discount* technique delivered moderate cost savings across all drugs. Its principal advantage lies in its simplicity and low administrative burden, as it does not require patient-level monitoring or outcome verification. This makes it a practical choice for high-cost drugs with well-established clinical efficacy but limited price flexibility [9,12].

### 4.2. Policy and Practice Implications

The findings of this study underscore that no single MEA technique is universally optimal; rather, the effectiveness of MEA depends on the dominant source of drug-related uncertainty. By explicitly linking real-world cost outcomes to uncertainty characteristics, this study provides a pragmatic framework to support more targeted and transparent MEA selection in market access decision-making.

In the Thai context, where full HTA processes are often resource-intensive and may delay reimbursement decisions for high-cost innovative drugs, MEAs can function as transitional market access instruments. They allow payers to manage financial risk while enabling earlier patient access under drug-related uncertainty conditions.

The results suggest differentiated policy strategies by uncertainty type. For drugs primarily characterized by *price uncertainty* (e.g., pertuzumab and osimertinib), financial-based MEAs such as free initiation treatment and utilization caps demonstrated substantial and predictable cost savings, making them suitable options for immediate budget risk mitigation. For drugs associated with *effectiveness uncertainty* (e.g., afatinib and ceritinib), MEA techniques that shift early treatment costs to manufacturers or link reimbursement to observed clinical benefit, particularly conditional treatment continuation, offer stronger alignment between payment and real-world performance. In contrast, drugs with predominant *use uncertainty* (e.g., palbociclib and ribociclib) benefit from MEAs that cap payer liability or condition continuation based on response, supporting greater budget predictability amid variable treatment durations.

From a system perspective, Thailand’s three major public health benefit schemes (CSMBS, SSS, and UCS) face increasing fiscal pressure from the growing use of high-cost anticancer drugs. The application of structured MEA frameworks, informed by dominant uncertainty types, may enhance consistency across schemes and reduce reliance on special or short-term price negotiations. However, effective implementation requires centralized governance, standardized MEA negotiation principles, and interoperable data systems capable of supporting utilization tracking and outcome monitoring. Strengthening these institutional foundations is essential for scaling MEAs beyond isolated agreements and embedding them within a sustainable national market access strategy.

In addition, although this study adopts a payer perspective, the practical implementation of MEAs ultimately depends on pharmaceutical companies’ willingness to participate in structured risk-sharing agreements. Performance-based agreements, such as conditional treatment continuation and pay-by-result, should not be interpreted as unilateral reimbursement restrictions; rather, they represent negotiated contractual mechanisms designed to align payment with observed clinical performance. In practice, these agreements are defined through mutual agreement on eligibility criteria, outcome measurement, monitoring procedures, and financial adjustment mechanisms.

Pharmaceutical companies may consider financial- or performance-based MEAs acceptable when such agreements facilitate earlier market access, reduce reimbursement uncertainty, and support long-term market sustainability. However, participation decisions are influenced by anticipated revenue impact, administrative complexity, data infrastructure requirements, and transparency expectations. Therefore, effective MEA implementation requires deliberate alignment of incentives between payers and pharmaceutical companies to ensure financial sustainability while preserving timely patient access [9]. By identifying the dominant uncertainty domains associated with different drug types, this study may also assist pharmaceutical companies in determining when specific MEA structures are strategically appropriate and mutually beneficial.

### 4.3. Sensitivity to Clinical Variability and Real-World Evidence

The sensitivity analyses highlight the importance of incorporating real-world clinical variability into MEA design. Changes in median PFS significantly affected cost savings for MEA techniques linked to treatment duration, underscoring the need for locally relevant real-world evidence when defining payer liability thresholds. Overreliance on trial-based estimates may lead to misaligned incentives and inaccurate projections of budget impact. Policymakers should therefore prioritize the integration of real-world data into MEA negotiations to ensure that agreements reflect actual treatment patterns and outcomes.

### 4.4. Limitations and Further Research

This study has several limitations that should be considered when interpreting the findings within a market access and reimbursement context.

First, the analysis was based on real-world data from a single tertiary-care hospital. Although TUH provides care under Thailand’s public health benefit schemes and reflects routine clinical practice for high-cost anticancer drugs, patient characteristics, treatment patterns, and discontinuation rates may vary across institutions and regions. Such variation could influence drug utilization and, consequently, the magnitude of cost savings achieved under different MEA techniques. As a result, the generalizability of the findings to other hospitals or to national-level implementation may be limited.

Second, the evaluation focused on five commonly applied MEA techniques, modeled individually for each drug to enable transparent comparison across dominant uncertainty types. More complex contractual agreements, such as hybrid MEAs that combine financial and performance-based agreements, or portfolio-based agreements covering multiple drugs, were not examined. Excluding these approaches may underestimate the full range of cost-containment strategies available to payers and limit the direct applicability of the results to more advanced market access negotiations.

Third, the analysis did not incorporate the administrative and operational costs associated with implementing and monitoring different MEA techniques. Performance-based agreements, in particular, require investment in data systems, outcome tracking, and contract management, which may offset part of the observed procurement cost savings. Without accounting for these implementation costs, the results reflect gross procurement savings rather than net economic impact from a system-wide perspective.

A further limitation relates to the scope of the economic evaluation. This study was designed as a cost-based analysis from the payer perspective and did not incorporate quality-of-life measurement or QALY estimation. While traditional cost-utility analyses are essential for comprehensive HTA decision-making, the present analysis focused on procurement cost implications and financial risk redistribution under alternative MEA structures. Future studies integrating MEA simulation with full cost-utility modeling may provide complementary insights.

Future research should therefore extend this work in several directions. Multicenter studies using national datasets that cover all major health benefit schemes would help validate the findings and improve external validity. Further analyses should also explore hybrid and portfolio-based MEAs to better reflect real-world contracting practices. Finally, incorporating administrative and transaction costs into economic evaluations would provide a more comprehensive assessment of the feasibility and net value of alternative MEA techniques in Thailand and similar healthcare systems.

## 5. Conclusions

This study provides real-world, policy-relevant evidence that aligning MEA techniques with dominant drug-related uncertainty characteristics can substantially enhance procurement cost savings for high-cost anticancer drugs. Using retrospective longitudinal utilization data and standardized scenario-based simulations, the analysis demonstrates that MEA performance varies systematically according to whether uncertainty is primarily related to price, effectiveness, or use.

Across all uncertainty types, the free initiation treatment technique achieved the greatest theoretical cost savings by shifting early treatment costs to pharmaceutical companies, particularly for drugs with uncertain real-world effectiveness. However, its implementation may be constrained by feasibility and negotiation considerations in routine market access practice. The conditional treatment continuation technique consistently ranked second, offering a more balanced approach that links reimbursement to observed clinical benefit while maintaining manageable administrative requirements. The discount and utilization cap technique provided moderate but predictable savings, while pay-by-result generated more limited savings under conditions of high response rates.

Taken together, these findings support the development of a structured, context-specific MEA framework for Thailand that moves beyond ad hoc price negotiations toward more transparent, uncertainty-informed market access strategies. By matching MEA design to the dominant source of reimbursement risk, policymakers can improve economic efficiency, support timely patient access, and strengthen the sustainability of public health financing for high-cost innovative anticancer drugs. The analytical approach presented in this study may also be applicable to other health systems facing similar challenges in managing uncertainty in the reimbursement of high-cost drugs.

## Figures and Tables

**Table 1:** Minimum treatment exposure thresholds applied for patient inclusion, based on indication-specific median PFS.

Drug	Primary Indication Used in This Study	Median PFS (Cycles)	Reference
Pertuzumab	HER2-positive MBC	17	[18]
Osimertinib	EGFR mutation-positive NSCLC	10	[19]
Afatinib	EGFR mutation-positive NSCLC	11	[20]
Ceritinib	ALK-rearranged NSCLC	16	[21]
Palbociclib	HR-positive, HER2-negative MBC	10	[22]
Ribociclib	HR-positive, HER2-negative MBC	10	[23]

Abbreviations: ALK, anaplastic lymphoma kinase gene; EGFR, epidermal growth factor receptor; HER2, human epidermal growth factor receptor 2; HR, hormone receptor; MBC, metastatic breast cancer; NSCLC, non-small-cell lung cancer; PFS, progression-free survival. Note: Median PFS values were derived from published studies corresponding to the primary indication most commonly observed in routine practice. These thresholds were applied solely to define minimum treatment exposure for retrospective cost modeling and MEA simulation and do not represent estimates of clinical benefit duration.

**Table 2:** Patient demographics.

	Price Uncertainty		Effectiveness Uncertainty		Use Uncertainty	
Parameters	Pertuzumab (*n* = 13)	Osimertinib (*n* = 66)	Afatinib (*n* = 9)	Ceritinib (*n* = 11)	Palbociclib (*n* = 23)	Ribociclib (*n* = 39)
Age, years Mean (SD)	59.23 (8.82)	70.67 (12.02)	71.56 (11.95)	61.27 (8.06)	65.04 (11.57)	62.56 (10.46)
Gender, *n* (%) Female Male	13 (100.00) 0 (0.00)	36 (54.55) 30 (45.45)	5 (55.56) 4 (44.44)	8 (72.73) 3 (27.27)	23 (100.00) 0 (0.00)	39 (100.00) 0 (0.00)
Health benefit scheme, *n* (%) CSMBS SSS UCS Others	9 (69.23) 0 (0.00) 3 (23.08) 1 (7.69)	54 (81.82) 0 (0.00) 10 (15.15) 2 (3.03)	5 (55.56) 0 (0.00) 3 (33.33) 1 (11.11)	8 (72.73) 1 (9.09) 2 (18.18) 0 (0.00)	12 (52.18) 2 (8.69) 6 (26.09) 3 (13.04)	24 (61.53) 1 (2.57) 13 (33.33) 1 (2.57)
HER2 expression status, *n* (%) Positive Negative	13 (100.00) 0 (0.00)				0 (0.00) 23 (100.00)	0 (0.00) 39 (100.00)
HR expression status, *n* (%) Positive Negative	4 (30.77) 9 (69.23)				0 (0.00) 23 (100.00)	0 (0.00) 39 (100.00)
EGFR mutation status, *n* (%) Positive Negative		66 (100.00) 0 (0.00)	9 (100.00) 0 (0.00)			
ALK expression status, *n* (%) Positive Negative				11 (100.00) 0 (0.00)		

Abbreviations: ALK, anaplastic lymphoma kinase gene; CSMBS, civil servant medical benefit scheme; EGFR, epidermal growth factor receptor; HER2, human epidermal growth factor receptor 2; HR, hormone receptor; SD, standard deviation; SSS, social security scheme; UCS, universal coverage scheme.

**Table 3:** Drug procurement cost savings across alternative MEA techniques for high-cost anticancer study drugs.

Scenario ^1^	Price Uncertainty						Effectiveness Uncertainty						Use Uncertainty					
	Pertuzumab			Osimertinib			Afatinib			Ceritinib			Palbociclib			Ribociclib		
	Cost per Patient (USD)	Total Cost (USD)	Cost Saving (%) ^2^	Cost per Patient (USD)	Total Cost (USD)	Cost Saving (%) ^2^	Cost per Patient (USD)	Total Cost (USD)	Cost Saving (%) ^2^	Cost per Patient (USD)	Total Cost (USD)	Cost Saving (%) ^2^	Cost per Patient (USD)	Total Cost (USD)	Cost Saving (%) ^2^	Cost per Patient (USD)	Total Cost (USD)	Cost Saving (%) ^2^
Reference case	43,281.83	562,663.77	–	77,386.07	5,107,480.50	–	15,923.05	143,307.41	–	28,413.30	312,546.26	–	39,396.38	906,116.78	–	19,005.24	741,204.24	–
1	30,297.28	393,864.64	30.00	38,693.03	2,553,740.25	50.00	7961.52	71,653.70	50.00	19,889.31	218,782.38	30.00	19,698.19	453,058.39	50.00	9502.62	370,602.12	50.00
2	14,162.07	155,146.26	72.43	32,431.12	1,899,884.05	62.80	3829.59	29,024.29	79.75	8118.08	64,741.72	79.29	17,690.73	363,601.00	59.87	8122.89	286,477.71	61.35
3	29,119.76	407,517.51	27.57	44,954.95	3,207,596.45	37.20	12,093.45	114,283.12	20.25	20,295.21	247,804.53	20.71	21,705.65	542,515.78	40.13	10,882.34	454,726.53	38.65
4	20,367.92	260,645.72	53.68	34,487.27	2,405,697.34	52.90	6046.73	59,862.59	58.23	17,047.98	178,597.86	42.86	18,067.13	432,858.33	52.23	6995.79	287,993.47	61.15
5	34,529.99	415,791.98	26.10	66,918.39	4,305,581.39	15.70	9876.32	88,886.87	37.97	25,166.06	243,339.59	22.14	35,757.86	796,459.34	12.10	15,118.69	574,471.18	22.49

^1^ The scenarios analyzed: Reference case, no MEA applied; Scenario 1, Discount technique; Scenario 2, Free initiation treatment technique; Scenario 3, Utilization cap technique; Scenario 4, Conditional treatment continuation technique; Scenario 5, Pay-by-result technique. ^2^ The difference in drug procurement cost between the reference case and after applying the MEA technique.

**Table 4:** Sensitivity analysis of drug procurement cost savings under changes in median PFS.

Scenario ^1^	Price Uncertainty						Effectiveness Uncertainty						Use Uncertainty					
	Pertuzumab			Osimertinib			Afatinib			Ceritinib			Palbociclib			Ribociclib		
	Cost Saving (USD)			Cost Saving (USD)			Cost Saving (USD)			Cost Saving (USD)			Cost Saving (USD)			Cost Saving (USD)		
	PFS − 10% ^2^	PFS	PFS + 10% ^3^	PFS − 10% ^2^	PFS	PFS + 10% ^3^	PFS − 10% ^2^	PFS	PFS + 10% ^3^	PFS − 10% ^2^	PFS	PFS + 10% ^3^	PFS − 10% ^2^	PFS	PFS + 10% ^3^	PFS − 10% ^2^	PFS	PFS + 10% ^3^
1	168,799.13	168,799.13	168,799.13	2,553,740.25	2,553,740.25	2,553,740.25	71,653.70	71,653.70	71,653.70	93,763.88	93,763.88	93,763.88	453,058.39	453,058.39	453,058.39	370,602.12	370,602.12	370,602.12
2	378,556.88	407,517.51	432,340.91	2,967,026.72	3,207,596.45	3,429,660.82	108,841.07	114,283.12	119,725.18	223,247.33	247,804.53	270,129.27	499,229.95	542,515.78	585,801.61	424,411.43	454,726.53	482,010.12
3	184,106.90	155,146.26	130,322.86	2,140,453.78	1,899,884.05	1,677,819.68	34,466.34	29,024.29	23,582.23	89,298.93	64,741.72	42,416.99	406,886.83	363,601.00	320,315.17	316,792.82	286,477.71	259,194.12
4	297,880.82	302,018.05	355,802.09	2,831,320.71	2,701,783.16	2,664,772.43	88,886.87	83,444.82	78,002.77	125,018.50	133,948.40	151,808.18	490,572.78	473,258.45	461,715.56	468,368.33	453,210.77	441,084.73
5	113,773.92	146,871.79	225,479.23	690,866.93	801,899.11	986,952.75	54,420.53	54,420.53	54,420.53	35,719.57	69,206.67	109,391.19	83,685.94	109,657.44	141,400.39	151,575.51	166,733.06	181,890.61

^1^ The scenarios analyzed: Scenario 1, Discount technique; Scenario 2, Free initiation treatment technique; Scenario 3, Utilization cap technique; Scenario 4, Conditional treatment continuation technique; Scenario 5, Pay-by-result technique. ^2^ The median PFS was decreased 10%. ^3^ The median PFS was increased 10%.

## Data Availability

The datasets presented in this article are not readily available because of ethical and privacy restrictions, as they contain sensitive patient-level information derived from the Thammasat University Hospital database. Access to the data may be granted upon reasonable request and subject to approval by the relevant institutional authorities.

## Abbreviations

The following abbreviations are used in this manuscript:

ALK	Anaplastic Lymphoma Kinase
CR	Complete Response
CSMBS	Civil Servant Medical Benefit Scheme
DMSIC	Drug and Medical Supply Information Center
EGFR	Epidermal Growth Factor Receptor
HR	Hormone Receptor
HTA	Health Technology Assessment
IOC	Item–Objective Congruence
MEA	Managed Entry Agreement
MBC	Metastatic Breast Cancer
NLEM	National List of Essential Medicines
NSCLC	Non-Small-Cell Lung Cancer
PAP	Patient Access Program
PFS	Progression-Free Survival
PR	Partial Response
RECIST	Response Evaluation Criteria in Solid Tumors
SD	Stable Disease
SSS	Social Security Scheme
TUH	Thammasat University Hospital
UCS	Universal Coverage Scheme
UHC	Universal Health Coverage
USD	United States Dollar
